# Durability of Adult Plant Resistance Gene *Yr18* in Partial Resistance Behavior of Wheat (*Triticum aestivum*) Genotypes with Different Degrees of Tolerance to Stripe Rust Disease, Caused by *Puccinia striiformis* f. sp. *tritici*: A Five-Year Study

**DOI:** 10.3390/plants10112262

**Published:** 2021-10-22

**Authors:** Ghady E. Omar, Yasser S. A. Mazrou, Mohammad K. EL-Kazzaz, Kamal E. Ghoniem, Mammduh A. Ashmawy, Amero A. Emeran, Ola I. Mabrouk, Yasser Nehela

**Affiliations:** 1 Agricultural Research Center, Wheat Diseases Research Department, Plant Pathology Research Institute, Giza 12619, Egypt; Dr_ashmawy2011@yhoo.com (M.A.A.); Ola_pathology@yahoo.com (O.I.M.); 2 Business Administration Department, Community College, King Khalid University, Guraiger, Abha 62529, Saudi Arabia; yasser.mazroua@agr.tanta.edu.eg; 3 Department of Agriculture Economic, Faculty of Agriculture, Tanta University, Tanta 31527, Egypt; 4 Agricultural Botany Department, Faculty of Agriculture, Kafrelsheikh University, Kafr Elsheikh 33516, Egypt; kelkazzaz@yahoo.com (M.K.E.-K.); Kamalghoniem@yahoo.com (K.E.G.); emeranaa@yahoo.com (A.A.E.); 5 Department of Agricultural Botany, Faculty of Agriculture, Tanta University, Tanta 31511, Egypt; 6 Citrus Research and Education Center, Department of Plant Pathology, University of Florida, 700 Experiment Station Rd., Lake Alfred, FL 33850, USA

**Keywords:** wheat, stripe rust, *Puccinia striiformis* f. sp. *tritici*, adult plant resistance (APR), *Yr18* gene, allelic variation

## Abstract

Adult plant resistance in wheat is an achievement of the breeding objective because of its durability in comparison with race-specific resistance. Partial resistance to wheat stripe rust disease was evaluated under greenhouse and field conditions during the period from 2016 to 2021. Misr 3, Sakha 95, and Giza 171 were the highest effective wheat genotypes against *Puccinia striiformis* f. sp. *tritici* races. Under greenhouse genotypes, Sakha 94, Giza 168, and Shandaweel1 were moderately susceptible, had the longest latent period and lowest values of the length of stripes and infection frequency at the adult stage. Partial resistance levels under field conditions were assessed, genotypes Sakha 94, Giza 168, and Shandaweel1 exhibited partial resistance against the disease. Leaf tip necrosis (LTN) was noted positively in three genotypes Sakha 94, Sakha 95, and Shandaweel1. Molecular analyses of *Yr18* were performed for *csLV34*, *cssfr1*, and *cssfr2* markers. Only Sakha 94 and Shandaweel1 proved to carry the *Yr18* resistance allele at both phenotypic and genotypic levels. Scanning electron microscopy (SEM) observed that the susceptible genotypes were colonized extensively on leaves, but on the slow-rusting genotype, the pustules were much less in number, diminutive, and poorly sporulation, which is similar to the pustule of NIL Jupateco73 ‘R’.

## 1. Introduction

Wheat (*Triticum aestivum* L.) is the most commonly grown cereal crop worldwide, and about one-third of the world’s population depends on it as a main source of food. In addition, it is an important source of protein, vitamins, minerals, and basic caloric value [1]. A new report by the USDA-Foreign Agricultural Service (FAS) in Cairo forecasts Egypt’s wheat production in 2020/2021 to reach 8.9 million metric tons (MMT), up by approximately 1.5% compared to 8.77 MMT in 2019/20. Moreover, in terms of imports, FAS Cairo forecasts Egyptian wheat imports in 2020/2021 at 12.85 MMT, up 0.4 percent from 2019/20 import [2]. However, this increased production is still not sufficient to provide food security for the ever-escalating population pressure. The productivity of wheat is of vital importance in the context of many biotic and abiotic factors that limit its production. Diseases and pests are the most important biotic factors affecting wheat productivity. Although wheat may be attacked by many phytopathogens and pests, about 20 diseases and five pests are of major significance. Some of these are spread all over the world, while others are limited to certain geographic districts or climatic zones [3]. Stripe rust was estimated to be the most currently important fungal disease in most wheat-growing areas [4].

Stripe rust of wheat is incited by the fungus *Puccinia striiformis* f. sp. *tritici* an obligate biotrophic pathogen that can disperse over long distances using wind-borne urediniospores [5]. The urediniospores, teliospores, and the hyphal stage on wheat are dikaryotic. Teliospores produce haploid basidiospores [6]. Pycnial and aecial spore stages of the fungus were confirmed recently on the alternate host *Berberis* spp. [7]. Historically, stripe rust has mainly been a problem in cool temperate regions; however, new races with adaptation to higher temperatures have been detected recently [8,9], suggesting the possibility of virulence changes in the pathogen population that are spreading rapidly and threatening wheat production worldwide [10].

Wheat production throughout the world is affected significantly by stripe rust. Regular rust epidemics have occurred and resulted in a significant decrease in numbers of kernels per head, lower kernel weights, and low grain quality [6,11]. The losses caused by the stripe rust can reach up 10–70%, depending on some factors. In severe cases, where infection occurs at early stages and disease development continues during the growing season, the yield loss reached 100% in susceptible genotypes. Until now, stripe rust on susceptible wheat cultivars is mainly controlled by fungicide applications [12]. However, their uses are undesirable due to health concerns, the hazard effects they inflict on the environment, and their high cost. In contrast, resistant cultivars are a potential, economical, and eco-friendly approach to controlling the disease.

In Egypt, the disease has overcome new resistant wheat cultivars only a few years after they were first used at a large scale in the field, causing grain yield losses to reach 69.33% [13]. With difficulties in the quick replacement of susceptible wheat cultivars, breeding cultivars with adequate levels of genetic resistance remains the most efficient method for controlling stripe rust as the long-term strategy [14]. It has been achieved by the deployment of cultivars with race-nonspecific resistance, also referred to as slow rusting, adult plant resistance, and partial resistance [15,16].

Adult plant resistance (APR) genes associated with slow rusting are believed to be more durable for successful long-term rust control in wheat. Wheat cultivars with APR may be susceptible at the seedling stage, but the expression of resistance increases at the flag leaf stage [17,18]. Slow rusting is a partial or incomplete resistance that permits the fungus to develop. It is generally not affected by the types of pathotypes, i.e., nonspecific in nature, and keeps the disease below the threshold level and decreases the chances of selection of new pathotypes [19,20]. In contrast, race-specific resistance provides a low infection type at the seedling stage and, most of the time, short-lived in nature due to the emergence of new virulences in the pathogen population to host selectivity in the field [21].

*Yr18/Lr34/Pm38/Sr57* is an important gene in wheat as it confers race-nonspecific, durable resistance to some diseases, including stripe rust, leaf rust, powdery mildew, and stem rust [22,23,24,25]. In addition, the expression of the *Yr18* gene induces leaf tip necrosis (LTN) phenotype in flag leaves of adult plants and likely involves senescence-like processes [26,27]. There is a great interest in developing effective methods for the detection of this gene. Some of the previous markers associated with *Yr18* were eliminated from application in breeding programs due to their low diagnostic ability across various wheat genotypes [28,29]. Newly developed markers that are closely linked to the *Yr18* locus have been shown to provide a widely diagnostic capacity in diverse wheat cultivar backgrounds [30,31]. The aim of the study was to characterize the durability of adult plant resistance gene *Yr18* in partial-resistance behavior of 13 wheat genotypes with different degrees of tolerance to *P. striiformis* f. sp. *tritici* during five successive seasons (from 2016 to 2021). We suggest that the presence or absence of the *Yr18* gene plays a key role in the durable resistance of wheat against *P. striiformis* f. sp. *tritici.* We used integrated epidemiological, phenotypic, and genotypic markers to better understand the partial-resistance behavior of wheat genotypes against variable levels of stripe rust infection. We believe that characterization of adult plant resistance of different wheat genotypes is a critical step to design better control guidelines/recommendations to minimize losses due to stripe rust disease. Moreover, it can be deployed to develop new resistant wheat cultivars.

## 2. Results

### 2.1. Sakha 95 and Misr 3, but Not Other Genotypes, Were Efficient against Pst Races at the Seedling Stage under Greenhouse Conditions

**Figure 1:**
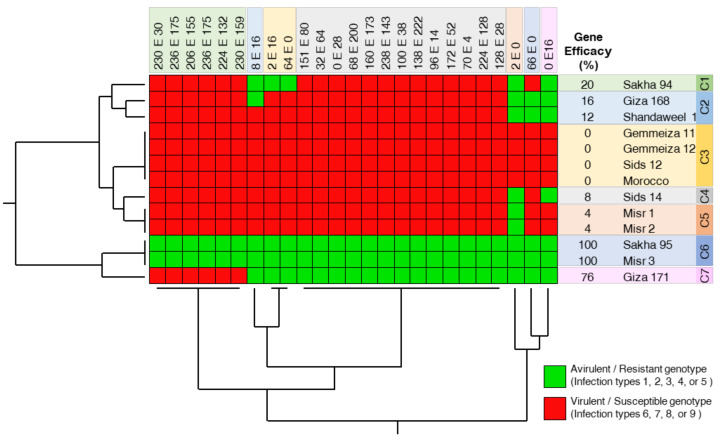
Efficacy of 13 selected wheat (*Triticum aestivum*) genotypes with different degrees of tolerance against stripe rust disease caused by *Puccinia striiformis* f. sp. *tritici* (*Pst*) at the seedling stage. Standardized two-way HCA based on the seedling response of each genotype against *Pst* races based on ITs as described by McNeal et al. [32]. The race was considered to be avirulent if it caused infection type 1, 2, 3, 4, or 5 (colored green) and considered to be virulent if it caused infection type 6, 7, 8, or 9 (colored red). Gene efficacy for stripe rust resistance was calculated by considering the number of susceptible responses over the total number of responses to the tested races. Two-way hierarchical cluster analysis (HCA) and its associated heatmap were visualized using JMP data analysis software, Version 15.

The efficacy of the tested wheat genotypes is shown in Figure 1. Briefly, most of the wheat genotypes were not efficient (efficacy ranged from 00.00% to 20.00%) against *Pst* races, except Sakha 95 and Misr 3, which had the highest efficacy (100%), followed by Giza 171 with 76.00% efficacy. The two-way hierarchical cluster analysis (HCA) showed that the 13 studied genotypes were arranged in approximately seven distinct clusters. Cluster 3 (C3) was the biggest cluster, which included four genotypes (Gemmeiza 11, Gemmeiza 12, Sids 12, and Morocco) with no efficacy at all against all tested *Pst* races at seedlings stages. On the other hand, cluster 6 (C6) included only two genotypes (Sakha 95 and Misr 3) with absolute efficacy against all tested *Pst* races at seedlings stages (Figure 1).

### 2.2. Assessment of Partial-Resistance/Slow-Rusting Components at Adult Stage under Greenhouse Conditions

**Figure 2:**
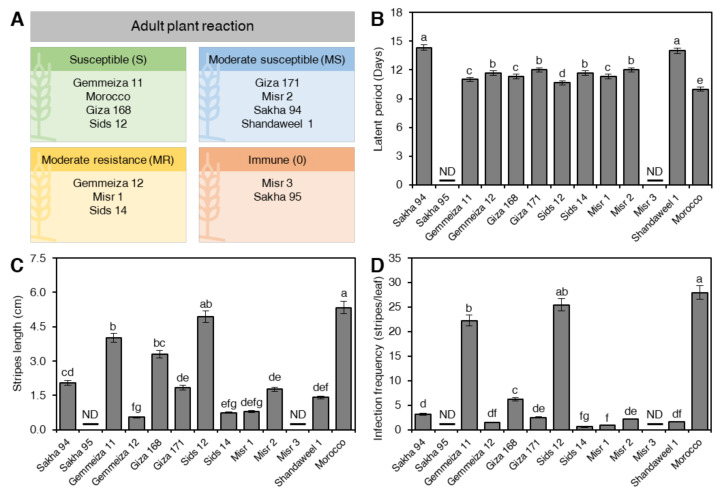
Components of partial resistance/slow rusting of 13 selected wheat (*Triticum aestivum*) genotypes with different degrees of tolerance against stripe rust disease caused by *Puccinia striiformis* f. sp. *tritici* (*Pst*) at adult stage under greenhouse conditions. (**A**) adult plant reaction, (**B**) latent period (Days), (**C**) stripes length (cm), and (**D**) infection frequency (stripes/leaf). Data presented are the means ± standard deviation (mean ± SD) of three biological replicates. Different letters indicate statistically significant differences between genotypes, whereas the same letters signify no significant differences between them based on Duncan’s multiple range test (*p* < 0.05).

The obtained data of partial-resistance (slow-rusting) components at the adult stage (Figure 2) indicated that significant variability was observed on the tested genotypes. Gemmeiza 11, Morocco, Giza 168, and Sids 12 showed susceptible (S) reactions with the highest percentages of rust severity. Moderately resistance reaction (MR) and the low percentage of rust severity was also observed on Gemmeiza 12, Misr 1, and Sids 14, while genotypes Giza 171, Misr 2, Sakha 94, and Shandaweel 1 showed moderate susceptible reaction (MS) with moderate rust severity (Figure 2A). Among the tested genotypes, only two genotypes, Misr 3 and Sakha 95, were completely resistant/immune (infection type 0, as assessed primarily according to the size of pustules and associated necrosis or chlorosis).

The shortest latent period was observed on Morocco, Sids 12 and Gemmeiza 11 (10.00, 10.66, and 11.00 days, respectively), while the longest latent period was recorded with Sakha 94, Shandaweel 1, Misr 2, and Giza 171 (14.33, 14.00, 12.00 and 12.00, respectively) (Figure 2B). Moreover, the stripes length (Figure 2C) and infection frequency (Figure 2D) differed significantly between all the genotypes; however, Shandaweel 1, Misr 2, Giza171, and Sakha 94 exhibited the lowest values of both components. On the other hand, the genotypes Giza 168, Gemmeiza 11, Sids12, and Morocco exhibited the higher values of both components. Lack of spore production was recorded in the case of genotypes Gemmeiza 12, Sids14, and Misr 1. However, no pustules were produced on Sakha 95 and Misr 3 (Figure 2C,D).

### 2.3. Partial Resistance under Field Conditions during the Period from 2016 to 2021

**Figure 3:**
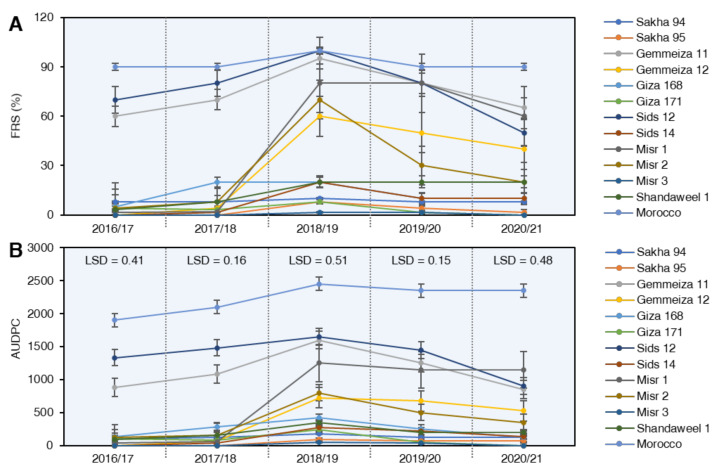
Partial-resistance parameters of 13 selected wheat (*Triticum aestivum*) genotypes with different degrees of tolerance against stripe rust disease caused by *Puccinia striiformis* f. sp. *tritici* (*Pst*) under field conditions during five successive seasons from 2016 to 2021. (**A**) final rust severity (FRS %), and (**B**) the area under disease progress curve (AUDPC). Values represent mean ± standard deviation (mean ± SD). LSDs (least significant difference) were calculated based on Duncan’s multiple range test (*p* < 0.05).

Epidemiological parameters of stripe rust disease, i.e., final rust severity (FRS %), the area under disease progress curve (AUDPC), rate of stripe rust increase (*r*-value), and relative resistance index (RRI), were detected during five successive growing seasons from 2016/17 to 2020/21 to determine the partial resistance of the selected wheat genotypes against *Pst* (Figure 3).

#### 2.3.1. Final Rust Severity (FRS %)

Final rust severity (FRS %) showed a range within the tested wheat genotypes and within growing seasons, as shown in (Figure 3A). Sakha 95 and Misr 3 proved to be highly resistant ones, which provided immune reaction during 2016/17 and 2017/18 seasons, and moderately resistant to stripe rust infection with low rust severity in 2018/19, 2019/20, and 2020/21 growing seasons. Giza 171, Sakha 94, Shandweel1, and Giza 168 genotypes slightly changed from moderately susceptible to susceptible response with low rust severities during the five growing seasons. Great changes were observed concerning Gemmeiza 12, Sids 14, Misr 1, and Misr 2, which showed resistant to moderately resistant infection type (low disease severity) during 2016/17 and 2017/18 seasons, and highly susceptible disease severity in 2018/19, 2019/20 and 2020/21 growing seasons (Figure 3A). In contrast, some wheat genotypes, i.e., Gemmeiza 11, Sids 12, and Morocco, showed inconstant results since they showed a highly susceptible response with the highest disease severities along the five growing seasons.

#### 2.3.2. Area under Disease Curve (AUDPC)

AUDPC was run in a parallel line with disease severity. The lowest values of AUDPC were recorded in Sakha 95 and Misr 3 during all of the growing seasons. Giza 171, Sakha 94, Shandweel1, Giza 168, and Sids 14 genotypes exhibited moderately AUDPC values, while the highest values of AUDPC were recorded with Gemmeiza11, Gemmeiza 12, Sids12, Misr 1, Misr 2, and Morocco during the five years of study (Figure 3B).

#### 2.3.3. Rate of Disease Increases (*r*-Value)

**Figure 4:**
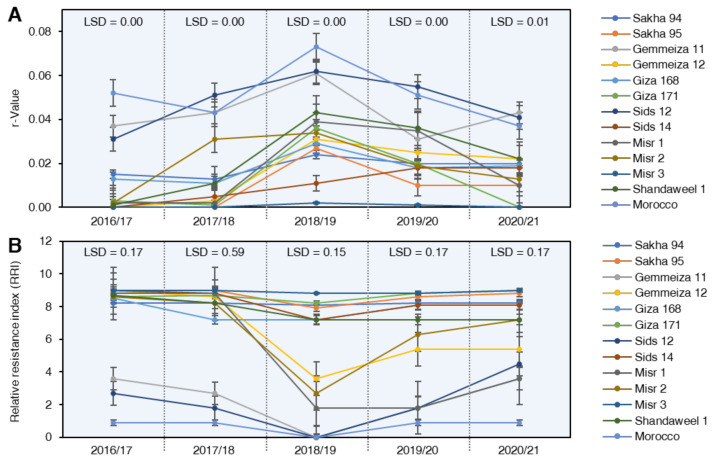
Partial-resistance parameters of 13 selected wheat (*Triticum aestivum*) genotypes with different degrees of tolerance against stripe rust disease caused by *Puccinia striiformis* f. sp. *tritici* (*Pst*) under field conditions during five successive seasons from 2016 to 2021. (**A**) rate of stripe rust increase (*r*-value), and (**B**) relative resistance index (RRI). Values represent mean ± standard deviation (mean ± SD). LSDs (least significant difference) were calculated based on Duncan’s multiple range test (*p* < 0.05).

Data concerning the rate of disease increases (*r*-value) showed that the wheat genotypes could be ranked into three groups: The first group included six genotypes, i.e., Sakha 95, Misr 3 Giza 171, Sakha 94, Shandweel1, Giza 168, and Sids 14, which exhibited stripe rust developed more slowly and increased at relatively lower rates of disease increase during the five growing seasons (Figure 4A). The second group included Gemmeiza 12, Misr 1, and Misr 2, which exhibited variable levels of *r*-values, starting with low *r*-values during the 2016/17 and 2018/19 seasons, then the *r*-values reached the maximum levels during the rest seasons and were classified as susceptible genotypes. The third group included the genotypes Gemmiza 11, Sids 12, and Morocco, which recorded a highly susceptible response to stripe rust infection, where the r-values reached the maximum level during all the seasons and were classified as fast rusting wheat genotypes (Figure 4A).

#### 2.3.4. Relative Resistance Index (RRI)

The frequency distribution of RRI values of the five-season trials for wheat genotypes is presented in (Figure 4B). All of the tested wheat genotypes showed desirable RRI to stripe rust except the three wheat genotypes, i.e., Gemmeiza 11, Sids 12, and Morocco, were placed under undesirable range during 2016/17 and 2017/18 growing seasons. While during the 2018/19, 2019/20, and 2020/21 seasons, the six wheat genotypes, i.e., Gemmeiza 11, Gemmeiza 12, Sids 12, Misr 1, Misr 2, and Morocco, were placed under undesirable range (Figure 4B).

#### 2.3.5. Observation of Phenotypic of APR Gene *Yr18*

**Figure 5:**
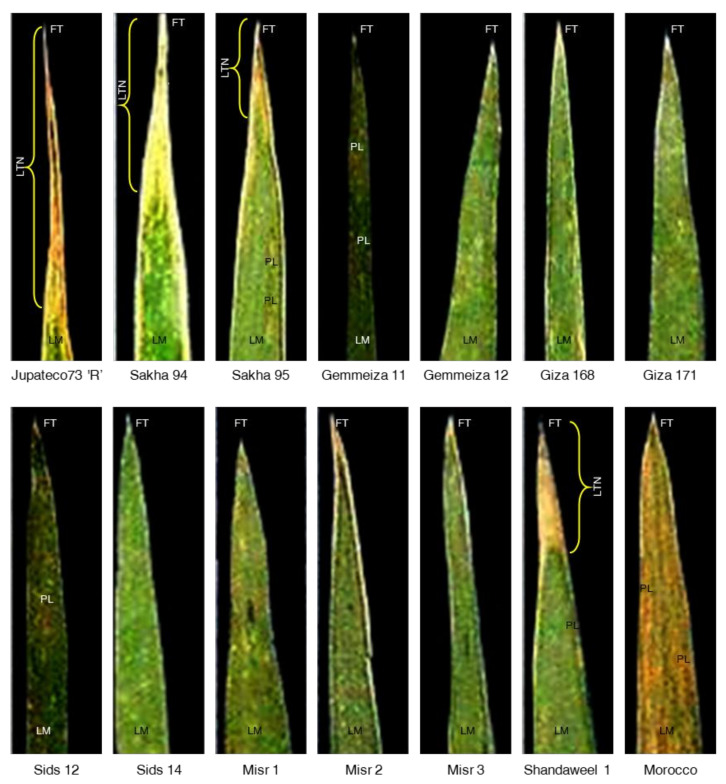
Leaf tip necrosis (LTN) displayed on the lamina of flag leaf of 13 selected wheat (*Triticum aestivum*) genotypes with different degrees of tolerance against stripe rust disease caused by *Puccinia striiformis* f. sp. *tritici* (*Pst*) compared with *Yr18* gene related to adult plant resistance in NIL Jupateco 73 ‘R’. LM: lamina, FT: forerunner tip (also known as Vorläuferspitze), LTN: leaf tip necrosis, and PL: elongated lesions of yellow-orange urediniospores erupting from pustules (the most characteristic symptom of stripe rust disease).

Leaf tip necrosis (LTN), a morphological trait, showed a complete linkage with the *Yr18* gene and could be used as a specific marker to identify wheat genotypes carrying this gene. Only three cultivars (Sakha 95, Sakha 94, and Shandaweel1), along with NIL Jupateco73 ‘R’, expressed LTN in the five growing seasons of the study, while the remaining 9 cultivars lacked LTN phenotype (Figure 5).

#### 2.3.6. Molecular Analyses to APR Gene *Yr18*

**Figure 6:**
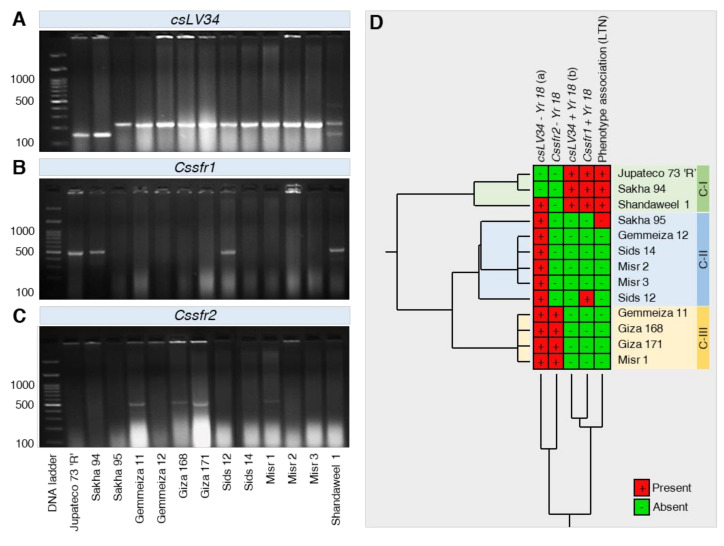
Amplification profile of the genotypic markers tagging the gene *Yr18* in 13 selected wheat (*Triticum aestivum*) genotypes with different degrees of tolerance against stripe rust disease caused by *Puccinia striiformis* f. sp. *tritici* (*Pst*) compared with *Yr18* gene related to adult plant resistance in NIL Jupateco 73 ‘R’. (**A**–**C**) Amplification profile of the genotypic markers *csLV34*, *Cssfr1*, and *Cssfr2*, respectively. (**D**) Standardized two-way HCA based on amplification profile of the genotypic markers (*csLV34*, *Cssfr1*, and *Cssfr2*) tagging the gene *Yr18*. Rows signify cultivars, while columns represent the genotypic markers. The presence of the genes is indicated by the plus sign (+) and colored red, whereas the absence of the gene is indicated by the minus sign (−) is colored green. Two-way hierarchical cluster analysis (HCA) and its associated heatmap were visualized using JMP Data analysis software-Version 15.

The STS marker *csLV34* was used and has shown polymorphism. This marker clearly represented resistant genotypes by producing 150 bp product size while genotypes amplifying 229 bp product size were susceptible for gene *Yr18*. Only positive control and Sakha 94 showed an amplified band of 150 bp, whereas 229bp band was amplified in 10 genotypes, i.e., Sakha 95, Gemmeiza 11, Gemmeiza 12, Giza 168, Giza 171, Sids 12, Sids 14, Misr 1, Misr 2, and Misr 3. However, Shandaweel 1 amplified the two bands 150 bp and 229 bp, as shown in Figure 6A. Gene-specific marker *cssfr1* showed a polymorphic profile with 517 bp product size, indicating the presence of *Yr18* gene in the only positive control, Sakha 94, Sids 12, and Shandaweel 1 (Figure 6B). Marker *cssfr2* amplified 52 bp fragments, indicating the absence of the *Yr18* gene in only four genotypes, namely, Gemmeiza 11, Giza 168, Giza 171, and Misr 1 (Figure 6C). Data of *csLV34* and *cssfr1* markers were corresponded to each other except for Sids 12, while data of *csLV34* and *cssfr2* markers corresponded to each other for genotypes, Sakha 94, Gemmeiza11, Giza 168, Giza 171, and Misr1. According to phenotypic and genotypic markers, Sakha 94 and Shandaweel1 proved to carry the *Yr18* gene (Figure 6D). The HCA analysis showed that both Sakha 94 and Shandaweel 1 genotypes were clustered together with the NIL Jupateco 73 ‘R’ (Cluster C-I) and separately from other cultivars (Figure 6D). It is worth mentioning that there were no differences among Gemmeiza 11, Giza 168, Giza 171, and Misr 1, which were clustered together at the bottom of the dendrogram with no evidence for the presence of the *Yr18* gene (Figure 6D).

## 3. Discussion

Stripe rust incited by *Puccinia striiformis* f. sp. *tritici* (*Pst*) has been a sporadic problem on wheat in Egypt, where epidemics arise mostly every decade. The disease has been consistently serious in the district during recent years. The rise of new virulent races of *Pst* can overcome the resistance constantly with the frequent replacement of wheat cultivars [33]. Knowledge of the identity of the stripe rust resistance genes in released cultivars is essential for the incorporation of new effective resistance genes into breeding programs and maintenance of a diversity of resistance genes in commonly grown cultivars. There is little information about adult plant resistance in Egyptian wheat cultivars, therefore, we carried out this study to determine the adult plant resistance (APR) in wheat cultivars at seedling and adult stages under greenhouse and field conditions during the period from 2016 to 2021.

In the current study, the efficacy of the tested wheat genotypes against the *Pst* races was evaluated at the seedling stage. The highest effective wheat genotypes were Misr 3, Sakha 95, and Giza 171, while the remaining cultivars had low efficacy against these races. It was evident that lacked seedling effective genes conferred resistance in wheat genotypes. These results are in contract with those informed by Zhang et al. and Pretorius et al. [14,34]. The absence of most of the effective seedling genes from commercial varieties prevents any forecasts of their response to aggressive stripe rust races.

At the adult stage, the results revealed that although all the tested genotypes were equally inoculated with the same *Pst* race under favorable environmental conditions, there was significant variation among them. The genotypes, Gemmeiza 11, Sids 12, and Giza168 showed the shortest incubation and latent periods and the highest disease severity, also recorded the highest length of stripes and infection frequency. Consequently, these genotypes could be classified as fast rusting. In contrast, the genotypes Sids 14, Misr1, and Gemmeiza 12 showed the lowest disease severity, moderate incubation period, shortest latent period, length of stripes, and infection frequency. In addition, the genotypes Sakha 95 and Misr 3 were free from infection; these genotypes could be categorized as resistant. While the genotypes Shandaweel 1, Giza 171, Misr 2, and Sakha 94 showed moderate susceptible responses and moderate values of the other trials, therefore, it could be characterized as slow rusting. A similar pattern was observed by [35]. Confirmation of these results [36] proves that the latent period was connected to the reaction type in which the cultivars with susceptible reaction types had shorter latent periods compared with those possessing resistant reactions.

Wheat genotypes responses under field conditions revealed high susceptibility to stripe rust during all the seasons of study. More stripe rust epidemic was recorded in the 2018/19 and 2019/2020 growing season than that in the previous two seasons, which the stripe rust epidemic was relatively lower. Among 13 genotypes tested, only three genotypes were moderately resistant to stripe rust during all the seasons, i.e., Sakha 95, Misr 3, and Giza 171. It was also observed that genotypes Gemmeiza 12, Sids 14, Misr 1, and Misr 2 rated resistant to moderately resistant in 2016/17 and 2017/18 seasons then lost their resistance in the third season 2018/19 showing highly susceptible response. On the contrary, Sids 12 and Gemmeiza 11 showed highly susceptible responses along the five seasons with the highest values of FRS %, AUDPC, r-value, and RRI. However, only three genotypes, Sakha 94, Shandaweel1, and Giza168, were distinct as possessing high levels of partial resistance according to the studied parameters (FRS %, AUDPC, r-value, and RRI) in five years of study. Based on the greenhouse and field data, genotypes Sakha 94, Giza186, and Shandaweel1 carry slow-rusting resistance at the adult stage. This type of resistance remains effective for a long time even if the pathogen changes its genotype [37]. These results run in the same trend with many other workers confirmed our finding in this respect [38,39].

It could be a challenge to avoid stripe rust epidemics in Egypt due to various wheat cultivars without genetic information that have been cultivated nationally. Therefore, identifying the resistance genes in wheat cultivars is important to explain the relationship between the host genetic pool and pathogen diversity. In the current study, stripe rust resistance gene *Yr18* was detected in Egyptian wheat genotypes.

*Yr18* is an important gene in wheat as it confers race-nonspecific, durable resistance to stripe rust and has been deployed for over 100 years [40]. The nucleotide sequence of *Yr18* spans 11,805 bp and consists of 24 exons, and is located on wheat chromosome 7DS [28]. Comparison of different wheat genotypes revealed two distinct haplotypes, a susceptible (−*Yr18*) and a resistant (+*Yr18*) haplotype. The previously haplotypes varied in three-nucleotide polymorphisms, two of which were located in exons, and only one single nucleotide polymorphism (SNP) was located in intron 4 [27]. Studies of Singh et al. [41] using the Jupateco73 ‘R’ near-isogenic reselections studies at CIMMYT have shown that the gene *Yr18* increases latent period at flag leaf stage and decreases infection frequency and length of infection lesions to stripe rust in greenhouse experiments. In addition, slow-rusting resistance based on *Yr18* protected grain yield depending on cultivar response and environmental condition, according to Ma and Singh, 1996 [42].

Postulating the presence or absence of the *Yr18* gene in genotypes based on adult plant reactions may be performed. However, this is complicated by the presence of other resistance genes and the effects of environmental variability on expression. Analysis of *Yr18* in genotypes based on LTN has been used as a marker to identify wheat genotypes carrying this gene. In the present study, LTN expression was observed positive in three genotypes Sakha 95, Sakha 94, and Shandaweel 1, along with positive control Jupateco73 ‘R’ in the five seasons, while the remaining 9 genotypes lacked LTN phenotype. Several observations were conducted about the molecular basis of LTN by Krattinger et al. [27], who noted that the LTN gene was induced in flag leaves of *Yr18* containing lines when compared to lines without the gene. Leaf tip necrosis was assessed by Kolmer et al. [40] to predict the presence of *Yr18* in 127 wheat genotypes at CIMMYT, Mexico. Only 52 lines lacked the LTN phenotype and possessed the non-*Yr18*-associated *csLV34*a allele, and about 75 lines expressed LTN, 62 from them carried the *Yr18*-associated *csLV34*b allele, while the remaining 13 lines had the *csLV34*a allele. Therefore, the background of the genotype and the multigenic effects on overall leaf tip necrosis expression can lead to equivocal results, and LTN is not equally expressed in all environments [26,43].

The importance of the adult plant rust resistance genes *Yr18* was confirmed via the use of tightly linked markers such as *csLV34*. Two alleles of *csLV34* were detected, included *csLV34a* (229 bp) and *csLV34b* (150 bp), which are associated with the susceptibility allele and the *Lr34* resistance allele, respectively [30]. In the present study, results revealed that the STS marker *csLV34* amplified 150 bp fragment in Sakha 94 and positive control, addressing the presence of *Yr18* gene, while 229 bp fragment was amplified in 10 genotypes and reported to be linked with the absence of *Yr18* gene. However, in Shandaweel 1 genotype, which is heterozygous, and both bands are generated simultaneously. *csLV34* marker, along with phenotypic analysis, was used to assume the presence of *Yr18* in wheat lines and cultivars [31,44,45]. Briefly, although the *csLV34b* allele was reported in some susceptible lines, a strong correlation was suggested between adult plant rust resistance and the presence of this allele, *csLV34b* [31,44,45].

In order to assess more accurately the presence of *Yr18* in the tested wheat genotypes, the newly developed gene-specific markers, *cssfr1* and *cssfr2,* which were developed based on these sequence differences between the resistance and susceptibility alleles, were used in this study [30]. Gene-specific marker *cssfr1* produced 517 bp in Sakha 94, Sids 12, Shandaweel 1, and in the positive control, suggesting the presence of the *Yr18* gene. Four genotypes, Gemmeiza 11, Giza168, Giza 171, and Misr1, showed 523bp fragments, indicating the absence of the *Yr18* gene according to the *cssfr2* marker. The two markers *csLV34* and *cssfr1* produced very nearly results, except for Sids 12 genotypes. Similar results were confirmed by Wu et al. [46] when evaluating some landraces were to stripe rust. These landraces were predicted to have *Yr18* based on the presence of the markers but had final disease severities higher than 70%. Such a gene may be under the influence of a suppressor. However, the results of markers *csLV34* and *cssfr2* correspond to each other for genotypes, Gemmeiza 11, Giza168, Giza 171, and Misr1.

All used markers revealed that the frequency of the *Yr18* gene is low in Egyptian wheat cultivars and needs to be increased to broaden the race-nonspecific resistance against stripe rust. In accordance with these results, the frequency of *Yr18* is extremely high in Chinese landraces at more than 80% that is much higher than that in other countries and regions worldwide [40]. The *csLV34* (b) allele was absent in most of the cultivars and was at low frequency in North Africa, the Middle East, and Southwestern Europe, also reported that in Egypt, the distribution of *csLV34* alleles was (8,1) a and b alleles, respectively. Disparate distribution at the *csLV34* locus between cultivars may be directly or indirectly caused by selection and breeding efforts aimed at joining *Yr18* into these genotypes.

The *Yr18* gene has been recently isolated and predicted to encode ABC-type proteins, which belong to the superfamily of ABC transporters that produce proteins connected to the plasma membrane. This type of protein plays an important role in transferring a wide range of materials in and out of the membrane. Cytotoxic macromolecules, including ions, can be transported against the diffusion gradient on both sides of the cell membrane [27,47,48]. Therefore, the mechanism of *Yr18* in providing relative resistance against stripe rust pathogen maybe remove toxins, metabolites, or more harmful substances released by pathogen into host cells; this makes the pathogen grow slowly with a long latent period and smaller pustules compared to the hosts, which do not possess this gene [49,50]. Thus, the deployment of durable resistance gene *Yr18* is an effective strategy to stripe rust control.

## 4. Materials and Methods

**Figure 7:**
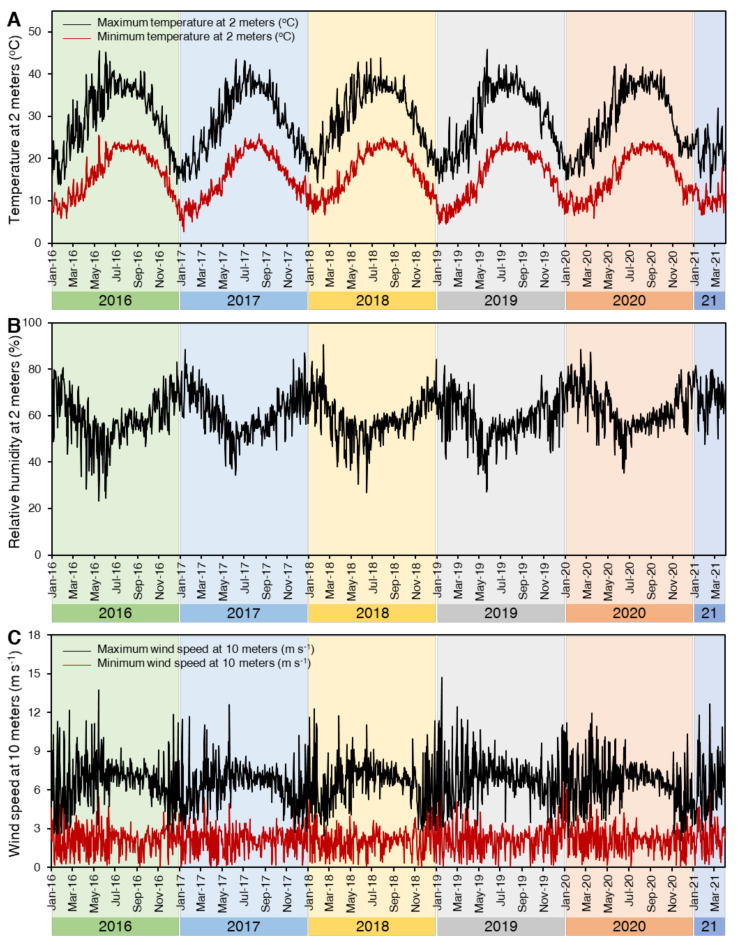
Meteorological data of the experimental site (31°5′57.4224″ N Latitude: 30°54′55.8288″ E Longitude) during 5-years growing seasons (2016–2020). (**A**) Temperature at 2 m (°C), (**B**) Relative humidity at 2 m (%), and (**C**) Wind speed at 10 m (m s^−1^). Presented data is based on the most recent available data on the Prediction of Worldwide Energy Resource (POWER; https://power.larc.nasa.gov/data-access-viewer/, accessed on 19 September 2021) Project, the National Aeronautics and Space Administration (NASA).

The present investigation was conducted at the greenhouse and experimental farm of stripe rust at Sakha Agriculture Research Station, Plant Pathology Research Institute (PPRI), Agricultural Research Center (ARC), Kafr El-Shaikh, Egypt (31°5′57.4224″ N Latitude, 30°54′55.8288″ E Longitude), during the period from 2016 to 2021, and Plant Pathology and Biotechnology Laboratory, Agricultural Botany Department, Faculty of Agriculture, Kafrelsheikh University, Egypt. Meteorological data of the experimental site during the 5-years growing seasons (2016–2021) are presented in Figure 7.

### 4.1. Plant Materials

In the current study, the durability of adult plant resistance gene *Yr18* in 12 wheat genotypes (Table S1) was investigated. These genotypes included Sakha 94, Sakha 95, Gemmeiza 11, Gemmeiza 12, Giza 168, Giza 171, Sids 12, Sids 14, Misr 1, Misr 2, Misr 3 and Shandaweel 1 along with NIL Jupateco73 ‘R’ (*Yr18* gene related to adult plant resistance) and universally susceptible check Morocco. All plant materials were obtained from Wheat Research Department, Field Crop Research Institute, ARC, Egypt.

### 4.2. Greenhouse Investigations

#### 4.2.1. Seedling Tests

Twenty five prevalent and aggressive races of *Puccinia striiformis* f. sp. *tritici* detected in Egypt from 2016 to 2020 namely 0E16, 128E28, 224E128, 66E0, 70E4, 172E52, 96E14, 64E0, 2E 16, 138E222, 100E38, 230E159, 2E0, 224E132, 238E143, 236E175, 160E173, 8E16, 68E200, 0E 28, 32E64, 151E80, 206E155, 236E175, and 230E30 were used to evaluate the seedling response of the above mentioned genotypes compared with the highly susceptible check Morocco. Briefly, eight-day-old seedlings of the tested genotypes were inoculated separately with the urediniospores of each race and then incubated in a dew chamber at 10 °C for 24 h. At 24 h post incubation (hpi), the inoculated seedlings were moved to the greenhouse, at 15 ± 2 °C, 80% ± 2% relative humidity, 8:16 h of light/dark period, and 7500 lux light intensity. Response of wheat seedling genotypes against different *Pst* races was recorded 10–15 days post inoculation (dpi) based on infection types (IT) as described by McNeal et al. [32]. Race was considered to be avirulent (resistant genotype) if it caused infection type 1, 2, 3, 4, or 5 and considered to be virulent (susceptible genotype) if it caused infection type 6, 7, 8, or 9. Gene efficacy for stripe rust resistance was calculated by considering the number of susceptible responses over the total number of responses to the tested races using Equation (1) according to the method adopted by Green [51] and Omara et al. [52].


(1) $$\mathrm{Gene efficacy (\%)}=\frac{\mathrm{Number of susceptible responses}}{\mathrm{Total number of responses to tested races}}\times 100$$

#### 4.2.2. Slow-Rusting Components at Adult Stage

*Pst* race 230E159, which was detected in 2017/18, was chosen to assess slow-rusting components at the adult stage during this year. A total of 10–15 wheat grains from each genotype were grown in a 25 cm diameter pot. Three biological replicates (pots) were used for each genotype and were inoculated with race 230E159 at the booting stage (corresponding to the phenological growth stages 41–45 on BBCH-scale) according to Tervet, 1951 [53]. After 24 h of incubation in dew chamber under less than 100% relative humidity and at 10 ± 2 °C, the inoculated pots were shifted to the greenhouse, where the temperature was 12 ± 2 °C under 80% relative humidity. The latent period (LP) (time from inoculation to pustule eruption) was measured according to Parlevliet [54]. The lengths of randomly chosen stripes on each leaf were measured [22]. Infection frequency was determined by measuring the infection lesions per unit leaf area (cm^2^) on the upper side of the leaves, as described by Parlevliet and Kuiper [55]. At the adult plant stage, infection type was assessed primarily according to the size of pustules and associated necrosis or chlorosis. At the adult stage, disease severity (DS%) was recorded on different genotypes, based on the percentage of leaf area covered with rust pustules. Field reaction of leaf rust infection types was classified into five categories: immune (0), resistant (R), moderately resistant (MR), moderately susceptible (MS), and susceptible (S) based on the scale described by Johnston [56] and Stakman et al. [57].

### 4.3. Field Investigations

The wheat genotypes under study were separately planted in adjacent rows within plots (3.5 × 3 m) in a randomized complete block design with three replicates. All plots were surrounded by a spreader area of one meter in width planted with the highly susceptible check Morocco. Artificial inoculation was relied on a mixture of virulent races of *Pst,* according to Tervet, 1951 [53]. Recommended agronomic practices, including fertilization dose and irrigation schedules, have been followed to maintain crop vigor. Briefly, phosphorus fertilizer (in the form of calcium superphosphate P_2_O_5_ 15% P) and potassium (in the form of potassium sulfate K_2_O 48% K) were added at the rate of 100 and 120 kg fed^−1^, respectively, during land preparation. Moreover, nitrogen fertilizer (in the form of ammonium nitrate 33.5% N) was applied at a rate of 300 kg fed^−1^ into three portions; 20% before planting, 40% before the second irrigation, while the remaining portion (40%) was applied before the third irrigation. Experimental plots were irrigated every 20–25 days as needed. All other agronomic practices such as hilling weeds, pests, and diseases management were conducted as recommended by the Ministry of Agriculture, Egypt.

#### 4.3.1. Disease Assessment

Data on disease severity were noted four times, every 10 day’s intervals during the five growing seasons 2016/17, 2017/18, 2018/19, 2019/20, and 2020/21. Two parameters were included during the scoring, i.e., severity according to the Modified Cobb Scale [58] and host reaction following Johnston, 1961 [56]. Epidemiological parameters expressed as the rate of stripe rust increase (r-value) and the area under the disease progress curve (AUDPC) were estimated using the formulas approved by Jeffers and Plank, 1965 and Pandey et al., 1989 [59,60]. The relative resistance Index (RRI) was calculated according to [61]. The standard for the desirable index was maintained at ≤7, whereas the value for the acceptable index was fixed as 6. The incidence or absence of leaf tip necrosis (LTN) was verified on flag leaves after the heading stage (corresponding to the phenological growth stages 54–56 on BBCH-scale) in the field during the five growing seasons [62].

#### 4.3.2. Molecular Analyses for *Yr18*

Genomic DNA was isolated from seedlings of the tested wheat genotypes along with positive control, NIL Jupateco 73 R following CTAB (Cetyl Tri-methyl Ammonium Bromide) extraction method as illustrated by Chen et al. [63]. Fresh leaves (60 mg) were freeze-dried, homogenized to a fine powder, allowed to thaw, and suspended in 1 mL of CTAB DNA isolation buffer. After thorough mixing, 1 mL chloroform/isoamyl alcohol was added, and samples were kept on a shaker for 20 min. The samples were then centrifuged at 12,000 rpm for 20 min, and the top aqueous layer of each sample was transferred to a new Eppendorf tube. The precipitate DNA was solved on 1 mL isopropanol then suspended in TE buffer. PCR amplification was performed in a 10 µL reaction using *csLV34, cssfr1,* and *cssfr2* primers (Table S2). The reaction mixture contained 1.0 µL (10 picomol) each of reverse and forward primers, 1.0 µL (2 mM) dNTPs, 1.0 µL 10× PCR buffer, 0.1 µL (5 unit.µL^−1^) Taq polymerase, 2.0 µL DNA (60–70 ng template DNA) and 4.9 µL d-H_2_O. PCR was performed in an Eppendorf f Mastercycler^®^ Gradient, at 94 °C for 3 min, followed by 45 cycles of 15 sec at 94 °C, 15 sec at 58 °C, 15 sec at 72 °C, and a final extension step of 10 min at 72 °C. PCR products were resolved by electrophoresis in 1.2% agarose gel and visualized under UV light following staining with ethidium bromide (500 µL.L^−1^).

### 4.4. Statistical Analysis

The trials were conducted in a randomized complete design with three replicates. All data were square-root transformed to ensure normal distribution of residuals. The analysis of variance of the data was performed with the software package SPSS 22. Duncan’s multiple range tests were used to compare treatment means [64]. Furthermore, two-way hierarchical cluster analysis (HCA) and its associated heatmap were used to visualize the seedling response against *Pst* races, as well as the amplification profile of the genotypic markers (*csLV34*, *Cssfr1*, and *Cssfr2*).

## 5. Conclusions

It could be concluded that the presence of variable levels of resistance among the Egyptian wheat genotypes, Sakha 94, Giza 168, and Shandaweel1 carrying slow rusting at the adult stage and may have APR resistance genes. Analysis of *Yr18* gene related to APR was performed, genotypes Sakha 94 and Shandaweel1 proved to carry *Yr18* based on phenotypic and genotypic markers. Growing of these cultivars is recommended to minimize losses due to stripe rust disease, and the *Yr18* gene can be deployed to develop new resistant wheat cultivars.

## Data Availability

The data that support the findings of this study are contained within the article or supplementary material and available from the corresponding author upon reasonable request.

## Supplementary Materials

The following are available online at https://www.mdpi.com/article/10.3390/plants10112262/s1, Table S1: Name and pedigree/selection history of wheat genotypes used in this study; Table S2: Marker name, sequences, PCR annealing temperature, expected product size, and reference used for the analysis of *Yr18* in selected wheat genotypes.
