# Supplementary material for: Durability of Adult Plant Resistance Gene Yr18 in Partial Resistance Behavior of Wheat (Triticum aestivum) Genotypes with Different Degrees of Tolerance to Stripe Rust Disease, Caused by Puccinia striiformis f. sp. tritici: A Five-Year Study
Source: Plants (Basel). 2021 Oct 22;10(11):2262. doi: 10.3390/plants10112262 (PMC8620947; doi:10.3390/plants10112262)
Supplement: Supplementary file 1 [file plants-10-02262-s001.zip › plants-1408034-supplementary.pdf]

Supplementary materials

Durability of Adult Plant Resistance Gene *Yr18* in Partial Resistance Behavior of Wheat (*Triticum aestivum*) Genotypes with Different Degrees of Tolerance to Stripe Rust Disease, Caused by *Puccinia striiformis* f. sp. *tritici*: A Five-Year Study

Ghady E. Omar [1,*], Yasser S. A. Mazrou [2,3], Mohammad K. EL-Kazzaz [4], Kamal E. Ghoniem [4], Mammduh A. Ashmawy [1], Amero A. Emeran [4], Ola I. Mabrouk [1] and Yasser Nehela [5,6,*]

[1] Wheat Diseases Research Department, Plant Pathology Research Institute, Agricultural Research Center, Giza 12619, Egypt; Dr_ashmawy2011@yhoo.com (M.A.A.); Ola_pathology@yahoo.com (O.I.M.)
[2] Business Administration Department, Community College, King Khalid University, Guraiger, Abha 62529, Saudi Arabia; yasser.mazroua@agr.tanta.edu.eg
[3] Department of Agriculture Economic, Faculty of Agriculture, Tanta University, Tanta 31527, Egypt
[4] Agricultural Botany Department, Faculty of Agriculture, Kafrelsheikh University, Kafr Elsheikh 33516, Egypt; kelkazzaz@yahoo.com (M.K.E.-K.); Kamalghoniem@yahoo.com (K.E.G.); emeranaa@yahoo.com (A.A.E.)
[5] Department of Agricultural Botany, Faculty of Agriculture, Tanta University, Tanta 31511, Egypt
[6] Department of Plant Pathology, Citrus Research and Education Center, University of Florida, 700 Experiment Station Rd., Lake Alfred, FL 33850, USA
* Correspondence: ghadyezzatomar@yahoo.com (G.E.O.); yasser.nehela@ufl.edu (Y.N.)

Table S1. Name and pedigree/selection history of wheat genotypes used in this study.

Genotypes	Pedigree
**Sakha 94**	OPATA/RAYON//KAUZ. CMBW90Y3180 0TOPM-3Y010M010M-010Y-10M-015Y-0Y-0AP-0S
**Sakha 95**	PASTOR // SITE / MO /3/ CHEN / AEGILOPS SQUARROSA (TAUS) // BCN /4/ WBLL1. CMA01Y00158S-040POY-040M-030ZTM-040SY-26M-0Y-0SY-0S.
**Gemmeiza 11**	BOW"S"/KVZ"S"//7C/SER182/3/GIZA168/SAKHA61GM58 20-3GM-1GM-2GM-0GM
**Gemmeiza 12**	OTUS/3/SARA/THB//VEECMSS97Y00227S-5Y010M-010Y-010M-2Y-1M-0Y-0GM
**Giza 168**	MAL / BUC // SERI. CM93046-8M-0Y-OM-2Y-0PMRL / BUC // SERI CM93046-8M-0Y-0M-2Y-0B
**Giza 171**	SAKHA 93/GEMMEIZA 9. S.6-1GZ-4GZ-1GZ-2GZ-0S
**Sids 12**	BUC//7C/ALD/5/MAYA74/ON//1160.147/3/BB/GLL/4/CHAT"S"/6/MAYA/VUL//CMH74A.630/4*SX. SD7096-4SD-1SD-1SD-0SD
**Sids 14**	Bow"s"/Vee"s"//Bow's'/Tsi/3/BANI SUEF 1. SD293-1SD-2SD-4SD-0SD
**Misr 1**	OASIS / SKAUZ // 4*BCN /3/ 2*PASTOR. CMSS00Y01881T-050M-030Y-030M-030WGY-33M-0Y-0S
**Misr 2**	SKAUZ / BAV92. CMSS96M03611S-1M-010SY-010M-010SY-8M-0Y-0S
**Misr 3**	ATTILA*2/PBW65*2/KACHU. CMSS06Y00582T-099TOPM-099Y-099ZTM-099Y-099M-10WGY-0B-0EGY
**Shandaweel 1**	SITE/MO/4/NAC/TH.AC//3*PVN/3/MIRLO/BUC. CMSS93B00567S-72Y-010M-010Y-010M-3Y-0M-0HTY-0SH
**Morocco**	Not available
**Jupateco 73 'R'**	(Yr18)II-12300//Lerma-Rojo-64/II-8156/3/Norteno-67[1281]; (S)Jupateco-73

Table S2. Marker name, sequences, PCR annealing temperature, expected product size and reference used for analysis of *Yr18* in selected wheat genotypes.

Marker Name	Marker Type	Sequence of Primers (5ˉ – 3ˉ)	Product Size (bp)	Annealing Temp. (C˚)	Reference
csLV34	STS	F: GTTGGTTAAGACTGGTGATGG<br>R: TGCTTGCTATTGCTGAATAGT	150<br>229	55	Lagudah *et al.,* (2006)
cssfr 1	SSR	F: TTGATGAAACCAGTTTTTTTTCTA<br>R: GCCATTTAACATAATCATGATGGA	517	58	Lagudah *et al.,* (2009)
cssfr2	SSR	F: TTGATGAAACCAGTTTTTTTTCTA<br>R: TATGCCATTTAACATAATCATGAA	523	58	Lagudah *et al.,* (2009)
